# Supplementary material for: Performance Evaluation of a Fully Automated Molecular Diagnostic System for Multiplex Detection of SARS-CoV-2, Influenza A/B Viruses, and Respiratory Syncytial Virus
Source: Diagnostics (Basel). 2025 Jul 16;15(14):1791. doi: 10.3390/diagnostics15141791 (PMC12294030; doi:10.3390/diagnostics15141791)
Supplement: Supplementary file 1 [file diagnostics-15-01791-s001.zip › diagnostics-3688028-supplementary.pdf]

Supplementary material of **Performance evaluation of a fully automated molecular diagnostic system for multiplex detection of SARS-CoV-2, influenza A/B viruses, and respiratory syncytial virus**

Supplementary Table

Table S1: Details of primers/probes and PCR conditions for manual RT-qPCR assays targeting SARS-CoV-2 *N* gene, IAV *M* gene, IBV *NS* gene, and RSV *M* gene

**Target virus**	**Primer/probe name and sequence 5′ – 3′**	**RT-PCR conditions**
SARS-CoV-2 [26]	NIID_2019-nCoV-N_F2<br>AAATTTTGGGGACCAGGAAC<br><br>NIID_2019-nCoV_N_R2<br>TGGCAGCTGTGTAGGTCAAC<br><br>NIID_2019-nCoV_N_P2<br>FAM-ATGTCGCGCATTGGCATGGA-TAMRA	50°C for 30 min<br>↓<br>95°C for 15 min<br>↓<br>[95°C for 15 s, 60°C for 60 s] × 45 cycles
IAV [25]	MP-39-67For<br>CCMAGGTCGAAACGTAYGTTCTCTCTATC<br><br>MP-183-153Rev<br>TGACAGRATYGGTCTTGTCTTTAGCCAYTCCA<br><br>MP-96-75ProbeAs<br>FAM-ATYTCGGCTTTGAGGGGGCCTG-MGB	50°C for 30 min<br>↓<br>95°C for 15 min<br>↓

		[94°C for 15 s, 56°C for 75 s] × 45 cycles
IBV [25]	NIID-TypeB TMPrimer-F1 5′-GGAGCAACCAATGCCAC-3′<br><br>NIID-TypeB TMPrimer-R1 5′-GTKTAGGCGGTCTTGACCAG-3′<br><br>NIID-TypeB Probe2 FAM-ATAAACTTYGAAGCAGGAAT-MGB	50°C for 30 min<br>↓<br>95°C for 15 min<br>↓<br>[94°C for 15 s, 56°C for 75 s] × 45 cycles
RSV [27]	Forward primer GGCAAATATGGAAACATACGTGAA<br><br>Reverse primer TCTTTTTCTAGGACATTGTAYTGAACAG<br><br>Probe FAM-CTGTGTATGTGGAGCCTTCGTGAAGCT-TAMRA	50°C for 10 min<br>↓<br>95°C for 10 min<br>↓<br>[95°C for 15 s, 58°C for 60 s] × 45 cycles

References

25. National Institute of Infectious Diseases, Influenza Diagnostic Manual, 5th Edition (In Japanese) Available online: https://id-info.jihs.go.jp/relevant/manual/010/influenza20230829.pdf (accessed on 11 July 2025).
26. National Institute of Infectious Diseases, Manual for the Detection of Pathogen 2019-NCoV Ver.2.9.1 Available online: https://id-info.jihs.go.jp/relevant/manual/010/2019-nCoV20200319.pdf (accessed on 11 July 2025).

27. National Institute of Infectious Diseases, Human Orthopneumovirus (RS Virus) Pathogen Detection Manual Version 4.0 Available online: https://id-info.jihs.go.jp/relevant/manual/010/RSVirus20230807.pdf (accessed on 11 July 2025).
